# Seven Months after Mild COVID-19: A Single-Centre Controlled Follow-Up Study in the District of Constance (FSC19-KN)

**DOI:** 10.1155/2022/8373697

**Published:** 2022-08-12

**Authors:** Elisabeth Haberland, Jonas Haberland, Stephan Richter, Michael Schmid, Julia Hromek, Heidi Zimmermann, Sabrina Geng, Hannes Winterer, Steffen Schneider, Marc Kollum

**Affiliations:** ^1^Hegau Bodensee Klinikum Singen, Gesundheitsverbund Landkreis Konstanz, Virchow Str. 10, Singen 78224, Germany; ^2^Landratsamt Konstanz, Amt für Gesundheit und Versorgung—Gesundheitsamt, Scheffelstraße 15, Radolfzell 78315, Germany; ^3^Institut für Herzinfarktforschung, Bremserstr. 79, Ludwigshafen 67063, Germany

## Abstract

**Objective:**

The primary aim of the study was to investigate the rate of hospitalization and admission diagnoses in severe acute respiratory syndrome coronavirus type 2 (SARS-CoV-2) positive patients seven months after initial infection. Secondarily, measurement of long-term effects on physical performance, quality of life, and functional outcome was intended.

**Design:**

The study is designed as a controlled follow-up of COVID-19 cases in the district of Constance (FSC19-KN). *Setting*. A controlled setting is provided due to the recruitment of an equally sized cohort consisting of age- and gender-matched subjects featuring similar cardiovascular risk profiles and negative SARS-CoV-2 antibody titers. *Participants*. The study examines 206 subjects after polymerase chain reaction (PCR) confirmed SARS-CoV-2 infection seven months after initial infection. *Exposure*. Infection in the SARS-CoV-2 positive group occurred between March and December 2020. *Main Outcome and Measures*. The frequency of inpatient admission during the observational period including the related diagnosis was defined as the primary endpoint. Secondary endpoints were health-related quality of life, physical performance, and functional outcome measured by European Quality of Life-5-Dimensions-5-Level (EQ-5D-5L), Short Form Health 36 (SF-36), Six-Minute Walk Test (6MWT), and Post-COVID-19 Functional Status (PCFS).

**Results:**

The study population consisted of mainly nonhospitalized subjects. During the first seven months of observation, frequency of inpatient admission was low and did not differ significantly between both groups (2.4% vs. 2.9% controls: OR 0.8, 95% CI 0.2 to 2.8). Calculation of six-minute walk distance ratios showed no significant difference between both cohorts (0.97 ± 0.17 vs. 0.98 ± 0.16 controls; mean difference −0.01; 95% CI −0.04 to 0.02). However, SARS-CoV-2-positive subjects achieved significantly lower EQ-5D-5L index scores (0.92 ± 0.12 vs. 0.95 ± 0.1 controls; mean difference −0.03, 95% CI −0.05 to −0.01) and SF-36 subscores. Reduced PCFS was reported significantly more often in the SARS-CoV-2 positive cohort (30.6% vs 14.6% controls: OR 2.6, 95% CI 1.6 to 4.2).

**Conclusion:**

The results suggest that mild COVID-19 has no impact on the hospitalization rate during the first seven months after infection. Despite unimpaired performance in cardiopulmonary exercise, SARS-CoV-2-positive subjects reported reduced quality of life and functional sequelae. Underlying psychoneurological mechanisms need further investigation. *Trial Registration*. This trial is registered with *clinicaltrials.gov* (identifier: NCT04724434) and *German Clinical Trials Register* (identifier: DKRS00022409).

## 1. Background

For over a hundred years, viral pandemics recurrently led to restrictions on public health. The Russian flu—possibly the very first coronavirus pandemic [1]—did not only cost the life of a million people at the end of the 19th century but also involved a prolonged convalescence in the surviving [2]. A more recent example is the secondary development of pulmonary damage in severe acute respiratory syndrome coronavirus (SARS-CoV) patients [3, 4]. Due to a similar taxonomic classification [5], it is possible that the characteristics mentioned above apply to the currently widespread SARS-CoV-2 as well.

The severity of coronavirus infectious disease 2019 (COVID-19) cases varies considerably. Courses are asymptomatic [6] or mild [7], most of the time without the necessity of hospitalization [8], but in some cases characterized by life-threatening acute respiratory distress syndrome (ARDS) [9]. Apart from the acute disease treatment, the clinical management of increasing numbers of post-COVID-19 patients [10] becomes challenging for emergency rooms and general practitioners. Their complaints are often unspecific [11], which makes the identification of secondary diseases a difficult task.

From a socioeconomic perspective, the management of post-COVID-19 patients needs to be planned efficiently on the basis of significant data. Since mild courses represent the majority of COVID-19 cases [12, 13], it is of particular interest to investigate sequelae in nonhospitalized individuals. Despite numerous follow-up studies, there remains a lack of convincing data in this field [14]. Sample sizes, age ranges, and study settings vary considerably, and data were rarely collected in a controlled setting [15]. Hence, the main goal of this single-center prospectivecontrolled follow-up study is to investigate clinical complications in a representative collective of COVID-19 cases.

## 2. Methods

### 2.1. Study Design

The prospective single-center cohort study FSC-19-KN was designed as a controlled follow-up of patients after SARS-CoV-2-infection in the local district of Constance (Baden-Wuerttemberg, Germany). Its main objective was to periodically assess sequelae over five years. Approval was given by the ethics committee of Albert Ludwigs University (Freiburg). The study was registered on the *German Clinical Trials Register* and *Clinicaltrials.gov*.

**Figure 1:**
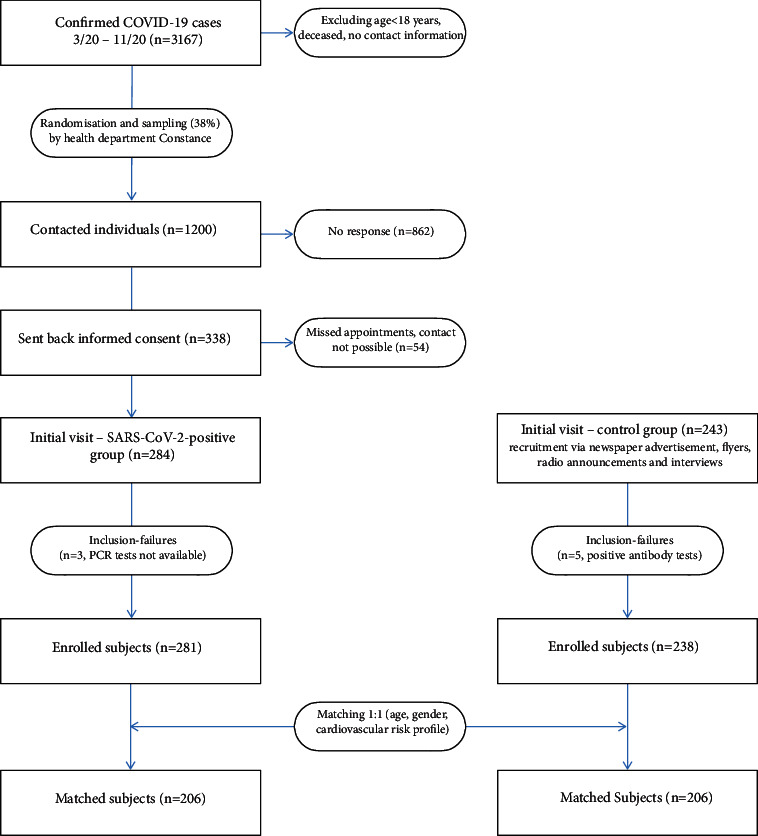
Recruitment and matching. Square boxes contain numbers of recruited subjects; round boxes contain numbers of excluded subjects.

The recruitment of the SARS-CoV-2-positive group was performed in cooperation with the local health department (see Figure 1). 1200 individuals were randomly sampled from all polymerase chain reaction (PCR)-confirmed cases in the local district of Constance between March 2020 and December 2020 and contacted via mail. During initial visits between January and July 2021, 281 adults who fulfilled the eligibility criteria were enrolled at a mean of 203.5 days after infection. Common eligibility criteria were defined as follows: age ≥18 years, ability to read and sign the consent form, and to grasp the nature of the study.

A total of 238 subjects exhibiting similar cardiovascular risk factors and negative SARS-CoV-2 antibody titers (Roche Elecsys Anti-SARS-CoV-2) were recruited as potential matching partners via newspaper advertisements, flyers, radio announcements, and interviews. 206 matching pairs could finally be established. Initially, the matching procedure followed strictly predetermined criteria (age ± 3 years, same gender, same status in terms of arterial hypertension, diabetes, and nicotine abuse). These were punctually loosened for 46 matching pairs in the following manner: age: ±5 years, both smoking and ex-smoking added together.

### 2.2. Data Collection and Outcome Measurement

All study data were collected in a clinical setting by the medical staff of the Hegau Bodensee academic teaching hospital (District of Constance, Germany). The supervising principal investigators worked as physicians in the field of internal medicine. The data were subsequently managed using a Research Electronic Data Capture (REDCap) platform hosted at redcap.glkn.de [16, 17]. The accuracy of data entries was verified by an external monitor according to guidelines for good clinical practice.

During initial visits, the medical history of the participants was recorded systematically with particular emphasis on COVID-19. This included the presence of symptoms, necessity of hospitalization, monitoring in an intensive care unit and mechanical ventilation, pre-existing medical conditions, and stratification of cardiovascular risk profile. Inpatient admissions within the previous seven months were inquired about to determine clinical events. Relevant medical reports were requested and evaluated. Admission diagnoses were referred to different medical disciplines. Since a greater effect of COVID-19 was expected on cardiopulmonary and neurological events [10, 18, 19], their frequencies were pooled and compared to those of the remaining surgical, gynecological, and orthopedic events.

The six-minute walk test was conducted in a clinical setting under the medical supervision of the principal investigators. Its protocol required documentation of Borg Categorial Ratio (CR) and Rating of Perceived Exertion (RPE) scales before and after physical stress. Ratios of six-minute walk distance were calculated with an age-, gender, and body mass index-dependant formula [20].

**Figure 2:**
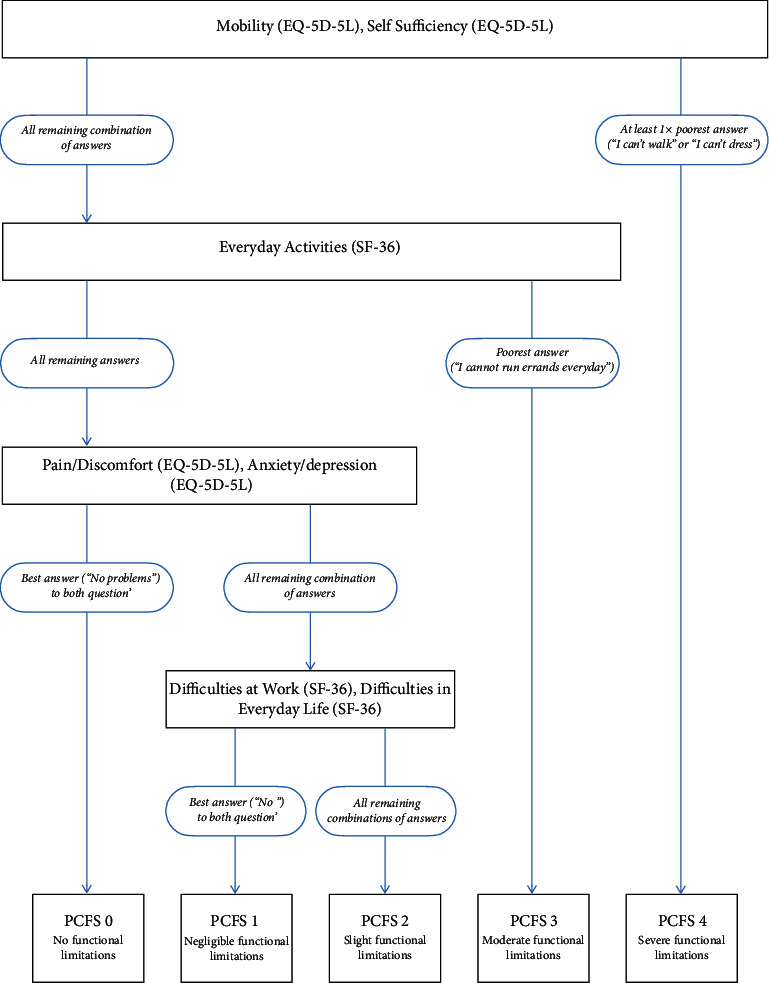
Translation algorithm from EQ-5D-5L and SF-36 into PCFS. Square boxes contain items from EQ-5D-5L/SF-36 and PCFS states; round boxes contain required response options.

Health-related quality of life was evaluated via German versions of European Quality of Life 5 Dimension 5 Level (EQ-5D-5L) and Short-Form Health 36 (SF-36). EQ-5D-5L index score was calculated using the German crosswalk value set according to the authors' algorithm [21] and the Visual Analogue Scale (VAS) score was directly read out. SF-36 subscores were calculated according to the authors' instructions [22]. Due to overlapping content, a translation algorithm from both questionnaires into the recently validated Post-COVID-Functional Status (PCFS) scale [23, 24] was developed and applied to generate further evidence for its clinical practicability (see Figure 2).

### 2.3. Statistical Analysis

Descriptive statistics were used for a comparative presentation of sociodemographic data, cardiovascular risk profiles, pre-existing medical conditions, and COVID-19-specific data. Results are presented only for matched data (n = 206, respectively). Discrete variables were presented as the absolute frequency with proportional value in brackets. Continuous variables were indicated as mean ± standard deviation. Missing values were recorded accurately, but not included during data analysis.

For Borg CR and RPE scales, six-minute walk distance ratios, EQ-5D-5L-index scores, SF-36 component, and subscores data were given as mean ± standard deviation. The difference in means (MD) was indicated and the respective 95%-confidence interval (CI) was estimated via *t*-test for independent samples with pooled variances. Results of PCFS were summarised into a fourfold table to calculate odds ratios (OR). Respective confidence intervals were determined by the logarithmic odds ratio function.

## 3. Results

### 3.1. Study Population

SARS-CoV-2-positive subjects were enrolled at a mean of 203.5 days after initial PCR testing. The majority had suffered from at least one symptom during the initial infection (95.1%). Rates of hospitalisation (2.4%), ICU-monitoring (0.7%), and mechanical ventilation (0.4%) were low.

The analysis of demographic and basic biometric data proved a similar composition of both study groups attributable to successful matching (Table 1). The mean age was 47 years with a moderately higher female participation rate of 58.2%, and body mass indices were at the threshold of mild obesity. The stratification of cardiovascular risk profiles showed no difference between both cohorts: 36% of participants were smokers, 13.1% suffered from arterial hypertension, and 1% from diabetes mellitus. Comorbidities were similarly prevalent in both cohorts with regard to chronic obstructive pulmonary disease, pulmonary embolism, deep venous thrombosis, myocardial infarction, cerebral ischemia, and coronary/peripheral artery disease. Merely bronchial asthma was found significantly more often in the SARS-CoV-2-positive cohort (8.9% vs. 3.0% controls: OR 2.5, 95% CI 1.1 to 6.0).

### 3.2. Primary Endpoints

Only clinical events leading to hospitalization were recorded (Table 2). They were distributed evenly among both study groups (2.4% vs. 2.9% controls: OR 0.8, 95% CI 0.2 to 2.8). Admission diagnoses were cardiopulmonary, neurological, orthopedic, gynecological, or surgical. Combined frequencies of cardiopulmonary and neurological events did not differ significantly between both groups (1.4% vs. 0.5% controls: OR 3.1, 95% CI 0.3 to 31.0).

### 3.3. Secondary Endpoints

Normal six-minute walk distance ratios were measured in both study groups (Table 3; means 0.97 ± 0.17 vs. 0.98 ± 0.16 controls) without a significant difference (MD −0.01, 95% CI −0.04 to 0.02). However, means were significantly higher in SARS-CoV-2-positive subjects' self-evaluation scores. This applied for the Borg CR scale at rest (0.05 ± 0.24 vs. 0.01 ± 0.08 controls; MD 0.04, 95% CI 0.01 to 0.09), after stress (0.8 ± 1.23vs. 0.41 ± 0.91 controls; MD 0.39, 95% CI 0.19 to 0.59), and RPE scale (9.6 ± 2.9 vs. 8.7 ± 2.6 controls; MD 0.8, 95% CI 0.3 to 1.4).

Regarding the EQ-5D-5L, means of both VAS (83.6 ± 15.2% vs. 88.6 ± 12.4% controls; MD −4.9, 95% CI −7.6 to −2.3) and calculated index scores (0.92 ± 0.12 vs. 0.95 ± 0.1 controls; MD −0.03, 95% CI −0.05 to −0.01) were significantly lower in the SARS-CoV-2-positive group.

Equally, SARS-CoV-2-positive subjects achieved significantly lower results in each of the eight SF-36 subscores (Table 3).

The majority of subjects altogether achieved lower PCFS states <2 (77.4%). However, reduced functional outcomes (PCFS ≥2) were detected significantly more often in the SARS-CoV-2-positive cohort (30.6% vs. 14.5% controls: OR 2.6, 95% CI 1.6 to 4.2).

## 4. Discussion

### 4.1. Interpretation of Results

Rates of hospitalization, monitoring in an intensive care unit, and mechanical ventilation were relatively low in the SARS-CoV-2-positive cohort. Most of the subjects were symptomatic but presented with a mild course of the disease.

No effect of SARS-CoV-2 infection could be detected on the frequency of clinical events during the first seven months after the initial infection.

Similarly, no significant effect of SARS-CoV-2 infection on physical performance during six-minute walk tests was observed. This harshly contrasts with poorer results in PCFS and Borg scales as well as EQ-5D-5L and SF-36 scores. Thus, the results suggest a negative impact of COVID-19 on functional outcomes and self-perceived quality of life.

### 4.2. Contextual Evidence

A huge number of recovered COVID-19 patients still suffer from symptoms six to eight months after mild infection [25, 26]. These symptoms are often unspecific and manifest in various organ systems [27], which makes it difficult to prove causality between prior SARS-CoV-2 infection and patients' complaints. According to a follow-up study carried out by the University of Ulm, there is a discrepancy between functional complaints and barely measurable organ damage in post-COVID-19 patients [28]. This resembles the contrast between poor self-evaluation and normal physical performance tests in the SARS-CoV-2-positive cohort at hand.

A large controlled follow-up study of COVID-19 patients in the United States investigated hospitalization rates six months after infection and provided evidence for higher incidences of pulmonary embolisms after COVID-19 [29]. Another equally scaled observational study from the same geographic region provided contradicting evidence stating that rates of thromboembolic events return to baseline pre-COVID-19 levels six to seven weeks after infection [30]. The SARS-CoV-2-positive cohort at hand showed no increase in thromboembolic events during the observational period.

The reduction of quality of life and functional outcome was reproduced by several other COVID-19 follow-up studies regardless of initial disease severity and cohort size [31–34]. Evidence has been published for postinfectious damage of neurons in the peripheral and central nervous system [35], which might contribute to the development of this very pattern. From a psychological perspective, mechanisms of social stigmatization [36] and the sheer knowledge of previous SARS-CoV-2-infection [37] should be additionally taken into account as harmful factors. A large multidisciplinary COVID-19 follow-up study initiated by University Mainz deals with this very subject by differentiating between knowingly and unknowingly infected subjects [37].

### 4.3. Limitations

First, the low hospitalization rate of the SARS-CoV-2-positive cohort seems to limitate the applicability of the findings. Since the severity of COVID-19 is mild in most cases [12, 13, 38], the study results are relevant for the majority of patients.

Second, the observational period has only been seven months so far. Long-term effects might not have been fully developed during initial visits yet. However, yearly follow-ups have been planned over the next five years to re-evaluate the results. Additional effects of SARS-CoV-2 vaccination and SARS-CoV-2 infection within the control cohort will have to be considered by then.

Third, there is growing evidence that postinfectious syndromes resemble psychiatric conditions such as fatigue syndrome and depression [39]. Mental symptoms have only been recorded to some extent via SF-36 subscores. However, a substudy has emerged from the study at hand dealing with this topic specifically.

Lastly, subclinical events requiring outpatient diagnostic and therapeutic procedures were not inquired about during initial visits. Considering the low frequency of clinical events so far, inclusion of these subclinical data is intended during future annual follow-ups.

## 5. Conclusion

In this study, SARS-CoV-2-positive subjects were mainly nonhospitalized and went through mild clinical courses. The initial data analysis suggests that there is no objective difference in terms of inpatient admission and physical performance between both study groups within the first seven months after infection. However, there is a pattern of negative self-assessment in the SARS-CoV-2-positive cohort with regards to health-related quality of life and functional status. Underlying psychoneurological mechanisms need to be investigated. During annual follow-up over five years, the dynamics of these effects will be monitored.

## Figures and Tables

**Table 1:** Study population.

	SARS-CoV-2, *n* = 206	Control, *n* = 206	Missing values, SARS-CoV-2/control
Demographics/biometrics			
Age (years)	47.0 ± 15.2	47.0 ± 15.0	*0/0*
18–39 (no. (%))	*69 (33.5)*	*63 (30.6)*	*0/0*
40 (no. (%))	*93 (45.1)*	*97 (47.1)*	*0/0*
60–79 (no. (%))	*43 (20.9)*	*46 (22.3)*	*0/0*
≥80 (no. (%))	*1 (0.01)*	*0 (0)*	*0/0*

Gender			
Male (no. (%))	*86 (41.8)*	*86 (41.8)*	*0/0*
Female (no. (%))	*120 (58.2)*	*120 (58.2)*	*0/0*

Body mass index (kg/m^2^)	*25.3* *±* *4.0*	*24.8* *±* *4.3*	*4/0*

Cardiovascular risk factors			
Diabetes mellitus (no. (%))	*1 (0.5)*	*1 (0.5)*	*0/0*
Arterial hypertension (no. (%))	*27 (13.1)*	*27 (13.1)*	*0/0*
Hypercholesterolemia (no. (%))	*24 (11.6)*	*15 (7.3)*	*2/0*
Smoking (no. (%))	*79 (36.9)*	*79 (36.9)*	*0/0*
Family history of coronary artery disease (no. (%))	*37 (18.9)*	*36 (17.5)*	*3/2*

Clinical history			
Chronic obstructive pulmonary disease (no. (%))	*3 (1.5)*	*2 (1.0)*	*10/6*
Interstitial lung disease (no. (%))	*0 (0.0)*	*0 (0.0)*	*9/7*
Pulmonary embolism (no. (%))	*1 (0.5)*	*1 (0.5)*	*8/7*
Deep vein thrombosis (no. (%))	*2 (1.0)*	*4 (1.9)*	*9/7*
Asthma (no. (%))	*18 (8.9)*	*8 (3.9)*	*22/12*
Myocardial infarction (no. (%))	*3 (1.5)*	*3 (1.5)*	*0/0*
Transient ischemic attack/stroke (no. (%))	*2 (1.0)*	*2 (1.0)*	*0/0*
Coronary artery disease (no. (%))	*4 (1.9)*	*4 (1.9)*	*0/0*
Peripheral artery disease (no. (%))	*1 (0.5)*	*1 (0.5)*	*1/0*

Data are given as absolute value (percentage)/mean ± standard deviation.

**Table 2:** Clinical events leading to hospitalization and admission diagnoses.

Total	SARS-CoV-2, *n* = 206 5 (2.4)		Control, n = 206 6 (2.9)	
Classification	Number	Admission diagnoses	Number	Admission diagnoses
Cardiopulmonal/neurological	3 (1.4)	Exclusion of coronary artery disease, atrial fibrillation, vestibular neuritis	1 (0.5)	Obstructive sleep apnoea

Orthopedic/surgical/gynecological	2 (0.9)	Tibial head fracture, meniscal lesion	5 (2.4)	Birth arrest, acute appendicitis, acute pancreatitis, meniscal lesion, spinal canal stenosis

Data are given as absolute value (percentage).

**Table 3:** Physical performance, health-related quality of life, and functional outcome.

	SARS-CoV-2 n = 206	Control n = 206	Difference in means/odds ratio	Missing values, SARS-CoV-2/control
6-minute walk-test results				
Walk distance (meters)	590.8 ± 77.7	600.8 ± 92.4	−10.4 *[ ****−****26.6; 6.5]*	*0/3*
Walk distance ratio (0-1)	0.97 ± 0.17	0.98 ± 0.16	−0.01 *[ ****−****0.04; 0.02]*	*4/3*

Borg scales				
Borg CR scale (0-10) at rest	0.05 ± 0.24	0.01 ± 0.08	***0.04 *** *[0.01; 0.09]*	*0/1*
Borg CR scale (0-10) after stress	0.8 ± 1.23	0.41 ± 0.91	***0.39 *** *[0.19; 0.59]*	*0/1*
Borg rating of perceived exertion (6-20)	9.6 ± 2.9	8.7 ± 2.6	***0.8 *** *[0.3; 1.4]*	*0/1*

EQ-5D-5L (European quality of life 5-dimension-5-level)				
VAS-index-score (%)	*83.6* *±* *15.2*	88.6 ± 12.4	***−4.9 *** *[ * ***−*** *7.6; −2.3]*	*0/0*
Calculated index-score (0-1)	*0.92* *±* *0.12*	0.95 ± 0.1	***−0.03 *** *[ * ***−*** *0.05; −0.01]*	*0/1*

SF-36 (short form 36) scores				
Physical functioning score (0–100)	*88.0* *±* *15.3*	93.6 ± 10.9	***−5.6 *** *[ * ***−*** *8.2; −2.9]*	*11/2*
Role functioning/physical score (0–100)	*76.0* *±* *33.9*	92.0 ± 22.7	***−16.0 *** *[ * ***−*** *21.6; −10.4]*	*1/0*
Role functioning/emotional score (0–100)	*73.6* *±* *37.8*	88.0 ± 27.0	***−14.4 *** *[ * ***−*** *20.8; −7.9]*	*10/3*
Energy/fatigue score (0–100)	*56.8* *±* *23.04*	67.9 ± 21.5	***−11.0 *** *[ * ***−*** *15.4; −6.7]*	*1/0*
Emotional well-being score (0–100)	*72.8* *±* *17.6*	81.1 ± 15.2	***−8.2 *** *[ * ***−*** *11.4; −5.0]*	*1/0*
Social functioning score (0–100)	*83.7* *±* *22.5*	90.7 ± 17.6	***−7.0 *** *[ * ***−*** *11.0; −3.1]*	*8/2*
Pain score (0–100)	*84.9* *±* *22.1*	90.7 ± 17.2	***−5.9 *** *[ * ***−*** *9.7; −2.0]*	*9/2*
General health score (0–100)	*72.8* *±* *21.0*	80.1 ± 16.9	***−7.3 *** *[ * ***−*** *11.0; −3.6]*	*0/0*

PCFS (post-COVID-19 functional status)				
PCFS 2/3 (no. (%)	*63 (30.6)*	30 (14.5)	*OR * ***2.6 *** *[1.6; 4.2]*	*0/0*

Data are given as mean ± standard deviation, and in square brackets, 95% confidence interval is given; significant differences in means/odds ratios are written in bold type.

## Data Availability

The data used to support the findings of this study are available in https://redcap.glkn.de/surveys/?__report = 9AKLDFRDCAER7AWF.

## Additional Points

Question: does a follow-up investigation of COVID-19 cases in a controlled setting show an increased hospitalization rate during the first seven months after infection? Findings: in this controlled follow-up study, the seven-month observation of 206 mildly affected SARS-CoV-2 positive subjects showed no significant increase in clinical events. Despite good performance in cardiopulmonary exercise, reduced quality of life and functional sequelae were reported. Meaning: long-term impact of mild COVID-19 does not manifest in binary endpoints such as hospitalization, but self-perceived health status.
